# Effect of Different Filler Wires on Mechanical Property and Conductivity of Aluminum-Copper Joints

**DOI:** 10.3390/ma13163648

**Published:** 2020-08-18

**Authors:** Hengming Zhang, Yu Shi, Yufen Gu, Chunkai Li

**Affiliations:** State Key Laboratory of Advanced Processing and Recycling of Non-ferrous Metals, Lanzhou University of Technology, Lanzhou 730050, China; zhm564145007@163.com (H.Z.); zzz61660512@163.com (Y.G.); wwj61660512@163.com (C.L.)

**Keywords:** different filler wires, alloy elements, microhardness, conductivity, tensile strength

## Abstract

The 1060 aluminum and T2 copper were joined by the pulsed double electrode gas metal arc welding (DE-GMAW) brazing method by using four types of filler wires, namely pure aluminum (Al) ER1100, aluminum-magnesium (Al–Mg) ER5356, aluminum-silicon (Al–Si) ER4043, and Al–Si ER4047, respectively. The effects of different types of filler wires on intermetallic compounds, microhardness tensile strength, and conductivity of joints were investigated. The results showed that a lot of brittle intermetallic compounds laying in the copper side brazing interface zone were generated using pure Al, Al–Mg, and Al–Si filler wires, which caused the change of microhardness, tensile strength, and the conductivity of joints. Meanwhile, with the increase in Si elements contents for Al–Sifiller wires, the thickness of the intermetallic compound layers decreased obviously, which was only up to 3 µm and the conductivity of the joints decreased. In addition, the microhardness, tensile strength, and the conductivity of the joints, when using Al–Sifiller wires, was higher than that using pure Al and Al–Mg filler wires. Hence, in comparison to the pure filler wires and Al–Mg filler wires, the Al–Si filler wires were more suitable for Al–Cu joints by DE-GMAW as Si element content was lower.

## 1. Introduction

Aluminum-copper (Al–Cu) dissimilar joints have many engineering applications due to their better conductivity, wetting, and ductility, such as the generator bus, conductive parts of transformer, refrigeration, aerospace, and chemical containers, etc. However, there are many challenges to joining Cu and Al because of the different physical properties, particularly thermal expansion coefficient, thermal conductivity, and melting point. Meanwhile, the formation of brittle intermetallic compounds (IMCs) can obviously deteriorate the mechanical properties of dissimilar joints [1,2,3,4].

In order to obtain the sound dissimilar metal joints with high reliability, good mechanical properties, and high conductivity, various welding methods have been developed. Yan et al. [5] investigated the influence of laser power on the microstructure and mechanical properties of laser-welded Al/Cu dissimilar lap joints and found that the shear strength of Al/Cu joints first increases and then, decreases with the increase in laser power; the maximum was about 99.8 MPa with the 2.45 kW laser power. Tian et al. [6] proposed the ultrasonic vibration-assisted friction stir welding (UVaFSW) technology, and it was found that ultrasonic vibration had a positive effect on the weld quality of joints; the value of tensile strength improves by 60.7% compared with that by FSW. N. Eslami et al. [7] realized the joining of aluminum-copper by friction stir welding, the results showing that the value of tensile strength was 110 MPa, which was similar to the aluminum-base material strength; the electrical resistance of the friction stir welds was between the electrical resistances of the base materials copper and aluminum. Emadinia et al. [8] adapted the magnetic pulse welding method to realize the bonding of dissimilar materials and found that the surface preparation determined the weld quality and intermetallic compounds appeared at the interface of some joints. C.W. Tan [9] obtained defect-free joints by friction stir welding technology and found that the presence of IMCs caused the distinct increase in hardness at the Al–Cu interface. Cai et al. [10] obtained sound Al–Cu joints using cold metal transfer technology and it was shown that the strength of the joints reached a higher level due to the effect of the size of Al–Cu IMCs. Feng et al. [11] joined aluminum to copper by CMT technology, the effect of heat input on the microstructure and tensile shear strength of joints was studied, and the results presented that different heat imputed resulted in the significant change of IMCs. Zhou et al. [12] adapted a low heat input pulsed double-electrode gas metal arc welding (DE-GMAW) brazing method [13] to achieve Al–Cu reliable connection, the intermetallic compound was controlled by heat input, and the sound joint was obtained.

Moreover, the effect mechanism of alloy elements from filler wires on the mechanical properties of aluminum-steel joints have been focused on by many scholars. Dong et al. [14] investigated the joining of aluminum-steel by flux-cored Zn-based filler wire and the alloy elements from filler wire had great effect on the tensile strength of the joints. Weigl M et al. [15] analyzed the aluminum-steel joints formed using Al–Si and Cu-Si alloys filler wires and it was found that the ductility of joints was significantly enhanced by using Al–Si alloy filler wires. Hong et al. [16] studied the aluminum-steel joints formed with Al–Si, Al–Cu, Al–Si–Cu, and Zn–Al filler wires, respectively. It found that the thickness of the IMC layer could be controlled as thin as about 2 μm and the tensile strength of the joint reached 136 MPa using Al–12% Si filler wire. 

However, there is little study about aluminum-copper joints formed with different filler wires; it is known that the different alloy elements from the filler wires have great effect on the formation of IMCs, and the mechanical properties and conductivity were determined by IMCs. Therefore, four filler wires consisting of different alloy elements were chosen and designed in this study, the DE-GMAW brazing method was adapted and the microstructure of the joint was analyzed by a low vacuum scanning electron microscope, then, the conductivity of the joint was tested by an intelligent metal conductor resistivity instrument. The aim of this study was to find the influence mechanism of alloy elements on the mechanical properties and conductivity of joints, which would provide guidance for selecting different filler wires. 

## 2. Experimental Procedures

The pure 1060 aluminum (CIMIC GROUP, Shanghai, China) and T2 copper (CIMIC GROUP, Shanghai, China) were prepared for base metal; the specimens all were 150 × 50 × 2 mm. ER1100, ER5356, ER4043, and ER4047 filler wires were used as filler materials and the diameter of filler wires was 1.2 mm, respectively. The chemical composition of 4 kinds of welding wires are shown in Table 1. The composition of base metal is shown in Table 2 and Table 3.

**Figure 1:**
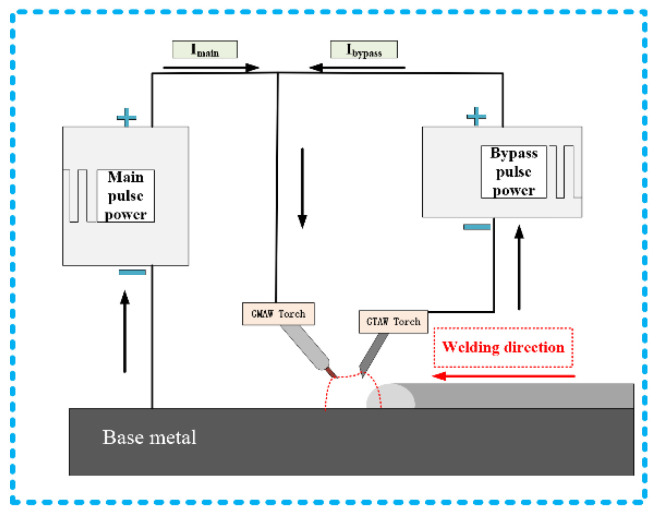
Schematic diagram of a welding system.

Figure 1 is the welding system. The DE-GMAW brazing method [17] was adopted and two pulsed power sources were used, which were MIG torch (RICHU Company, Shanghai, China) and TIG torch (RICHU Company, Shanghai, China), respectively. The main road current was utilized to molt the filler wires; it consisted of two parts: one was flow to the base metal, another part was flow to the GTAW torch (RICHU Company, Shanghai, China). The current in the welding circuit had the following relationship, as shown as Equation (1): I was the total current, $I_{bp}$ was the bypass road current, $I_{bm}$ was base metal current. Owing to the diversion effect of the bypass gas tungsten arc welding (GTAW) torch, it was beneficial to control the intermetallic compound layers with the decrease in heat input of the base metal.

(1) $$I_{bm}=I-I_{bp}$$

In this experiment, Table 4 shows the welding parameters, using argon as a shielding gas in the experiment, the argon gas flow was 20 L/min in the main, and in the bypass, the argon gas flow was 5 L/min. Average current was set 50/60/70 A in the main road ($I_{main}=50\;A\backslash 60\;A$\70 A), the average current was 25 A in the bypass road. Synchronous pulse frequency acting on the main and the bypass was 80 Hz, and duty cycle was all set as 15% for the main and the bypass. The welding speed was set as 0.6 m/min.

**Figure 2:**
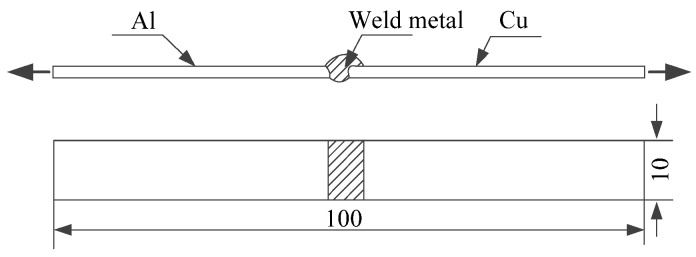
Dimensions of the tensile test sample (unit: mm).

Several sets of experiments were carried out with different filler wires, which were ER1100, ER5356, ER4043, and ER4047, respectively. Satisfactory butt joints were obtained ultimately ($I_{main}=60\;A$). The gap of the butt joint was zero. The surface of the butt joint was polished with a wire brush in order to ensure the flatness of the surface of the butt joint before the experiments. Simultaneously, in order to decrease the influence of oil contamination, the surfaces of the specimens were necessarily wiped with acetone before welding; it results that the surface is no water and oil. The microstructure of the joint in different filler wires was observed and analyzed by a low vacuum scanning electron microscope (SEM, Jeol company, Tokyo, Japan) of JSM-5600LV and microhardness was measured using a microhardness tester (ZHONGTE Precision Instrument Technology Company, Guangdong, China) of HVS-1000Z. When the width of the specimens was to 10 mm, as shown in Figure 2, an electronic tensile testing machine was selected to analyze the change in tensile strength.

**Figure 3:**
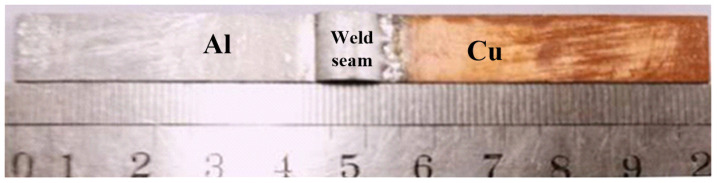
The specimen for testing conductivity.

As shown in Figure 3, in order to test the conductivity of the butt joints, which were cut 100 mm long × 10 mm width × 3 mm thick. In this paper, the TX-300A intelligent metal conductor resistivity instrument (China) produced by Xiamen Tianyan Instrument limited company (as shown in Table 5) was used to test the conductivity of the joint. The conductivity value was measured five times and the average to guarantee the accuracy of the measured value was calculated. 

## 3. Results

### 3.1. Cross Section of Joints

**Figure 4:**
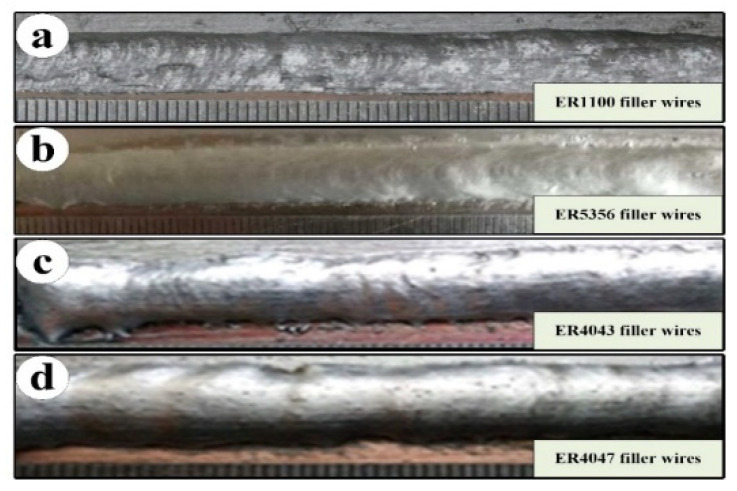
Surface appearances of joints at different filler wires ($I_{main}=60\;A$). (**a**) ER1100 filler wires; (**b**) ER5356 filler wires; (**c**) ER4043 filler wires; (**d**) ER4047 filler wires.

The sound joints were obtained by the pulsed DE-GMAW technology, and they were composed of melting filler wires, aluminum base, and copper base, but copper base was hardly molten. Shown in Figure 4, the melting filler wires were spread out on the surface and formed the brazing joints.

**Figure 5:**
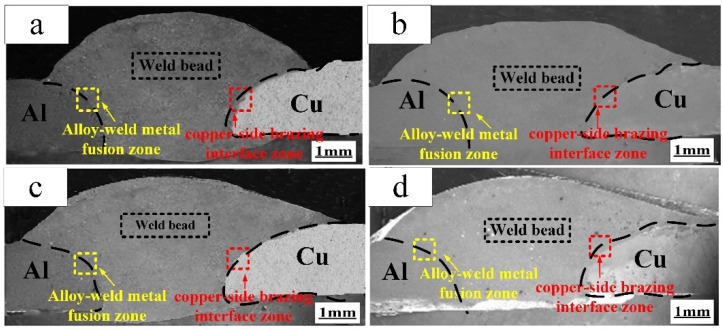
Cross section of the butt joints ($I_{main}=60\;A$). (**a**) ER1100 filler wires; (**b**) ER5356 filler wires; (**c**) ER4043 filler wires; (**d**) ER4047 filler wires.

The macrostructure of joints was obtained by using different filler wires, and the typical joints were selected as shown in Figure 5. It is evident that that cross section of joints consisted of an alloy-weld metal fusion zone, weld zone, and copper side brazing interface zone. However, comparing the aluminum side, it could be seen that joints failure always be occurred in the copper side brazing interface zone, and the alloy elements from filler wires mainly appeared at the copper side brazing interface zone, which caused the microstructure of the joints to change. 

### 3.2. Microstructure of the Butt Joints

**Figure 6:**
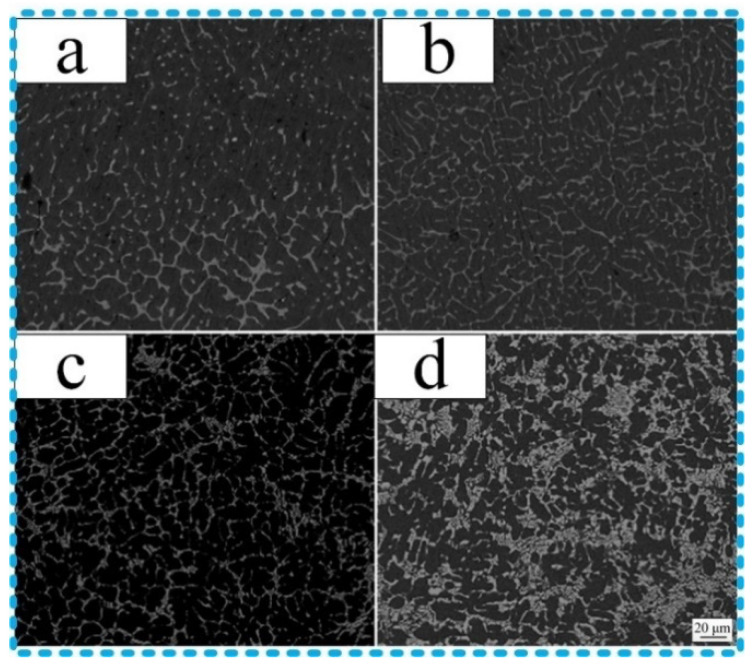
Microstructure of the butt joints (*I_main_* = 60 A). (**a**) ER1100 filler wires; (**b**) ER5356 filler wires; (**c**) ER4043 filler wires; (**d**) ER4047 filler wires.

According to the Table 1, the alloy elements from filler wires increased and bringing to the butt joints, it caused the amount of eutectic structure to become larger than that after cooling. Figure 6 shows the microstructure of the butt joints in different filler wires; it was observed from Figure 6a–c, the results show that microstructure of the butt joints consisted of α(Al) solid solution and net-shaped Al–Cu eutectic when using ER1100, ER5356, and ER4043. Yet, by using ER4047 filling wires, it was composed of coral-shaped Al–Cu eutectic, as shown in Figure 6d.

### 3.3. Microstructure of Copper Side Brazing Interface Zone

According to the references [1,2,3,4], the copper side brazing interface zone plays an important role to analyze mechanical properties, where the fracture failure always appeared. Therefore, it had great significance to analyze the microstructure of the copper side brazing interface zone formed by different filler wires. Figure 6 displays the microstructure of the copper side brazing interface zone, which was fed by ER1100, ER5356, ER4043, and ER4047 wires, respectively. It can be subdivided into the intermetallic compound layer and α-Al/Mg solid solution; the intermetallic compound was mainly concentrated in the copper side brazing interface zone and determined the mechanical properties. Following energy dispersive spectrometer (EDS) analysis and the results as shown in Table 6, referring to the binary phase diagram and correlative, it was demonstrated that the contents of the intermetallic compounds obtained by four filler wires were almost the same, which were mainly composed of Al_2_Cu; the Al–Cu eutectic area consisted of α phase and Al_2_Cu. While it was found that an extraordinarily solid solution of the joints contained a little Mg element using Al–Mg ER5356 filler wires, besides, there were few Si elements in the solid solution of the joint with Al–Si filler wires (ER4043 and ER4047). Just for ER4047 Al–Si filler wires, the bulk Si elements were precipitated in the joint of the Al–Cu eutectic area. The alloy elements were introduced from different filler wires and obviously impacted on mechanical and conductivity.

**Figure 7:**
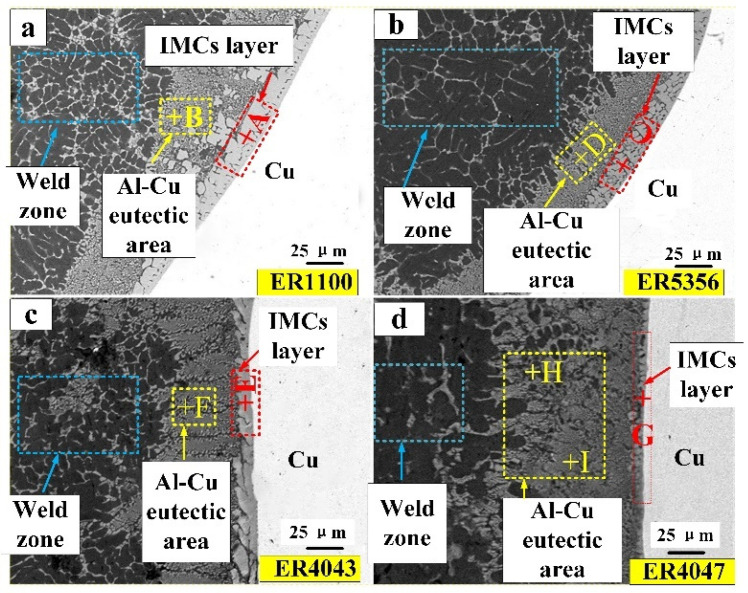
Microstructure of joints at copper side brazing interface zone (*I_main_* = 60 A): (**a**) ER1100 welding joint; (**b**) ER5356 welding joint; (**c**) ER4043 welding joint; (**d**) ER4047 welding joint.

It can be observed from Figure 7 that by using the ER1100 pure aluminum filler wire, the thickness of the intermetallic compound layer was about 20 µm, but for ER4047 aluminum alloy filler wires, thickness was 3 µm. Table 6 shows that the content of Si in the ER4047 and ER4043 aluminum alloy filler wires was approximately 12% and 5%, respectively, and Si was the main alloy element. Yet, for ER5356 filler wires, Si was only 0.25% and Mg content was 5%; Mg became the main alloy element.

### 3.4. Tensile Strength Test

The tensile strength of the joints was focused and analyzed by testing; the value of the tensile strength of the joints was found as shown in Table 7. Due to the effect of alloy elements from different filler wires, the tensile strength of the joints can be changed. The results show that the tensile strength for all joints made with Al–Si filler wires measured higher than 150 MPa, while that with Al–Mg filler wires was as low as only 112 MPa. As using the ER1100 pure aluminum filler wires, the formality of the weld seam was the worst and the testing of tensile strength was not able to be performed. It was observed from Table 7 that the average tensile strength was higher when the main alloy elements were Si than that when the main alloy elements were others.

### 3.5. Microhardness Test

**Figure 8:**
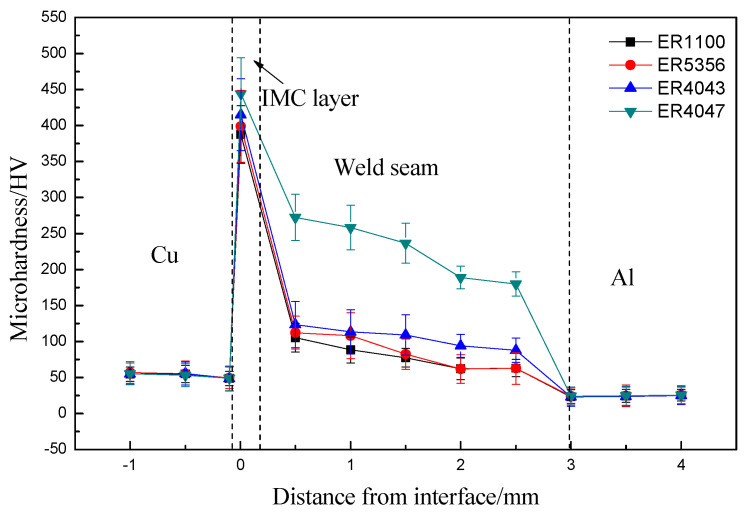
Microhardness distribution of the butt joints.

To further investigate the effect of alloy elements on microhardness, the microhardness of the copper side brazing interface zone was analyzed. Figure 8 shows the microhardness distribution of the copper side brazing interface zone. The copper base was seen on the left, there was aluminum base on the right side, and the butt joints were located in the middle. It was observed that the microhardness of copper base was higher than that of aluminum base, but it was lower than that of the butt joints. For the butt joints, it can be concluded that microhardness was the highest using ER4047 filler wires especially.

### 3.6. The Conductivity Test

The conductivity of base metal was obtained by testing as shown in Table 8. The resistivity of pure aluminum and T2 copper was 2.84 × 10^−8^ and 1.65 × 10^−8^, respectively. Table 9 shows the physical and mechanical properties of pure aluminum and T2 copper obtained from references [18,19]. It can be seen that the conductivity obtained by testing reached the same conductivity level found by the references.

Additionally, the conductivity of the joints formed using ER1100, ER5356, ER4043, and ER4047 filler wires was measured, and the test results are shown in Table 10. The results show that average current in the main road and the types of filler wires determined the conductivity and resistivity of the joints. The effect mechanism is explained in the next discussion part.

**Figure 9:**
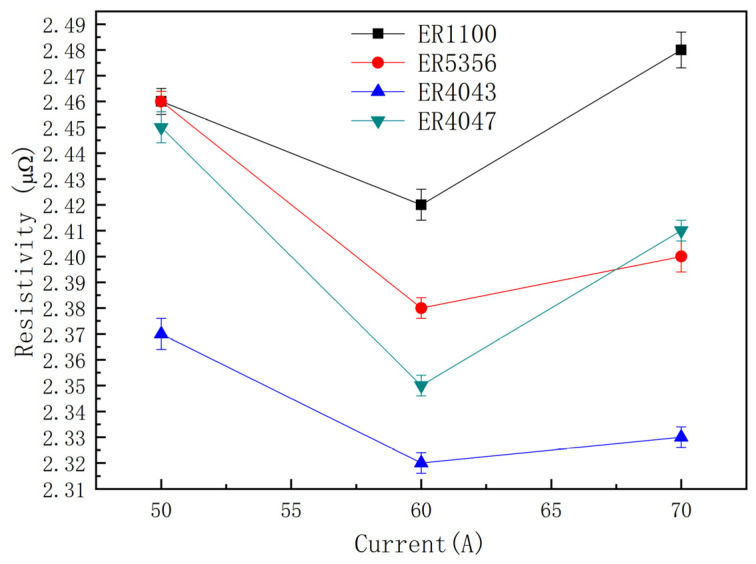
Resistivity of joints in different heat input.

**Figure 10:**
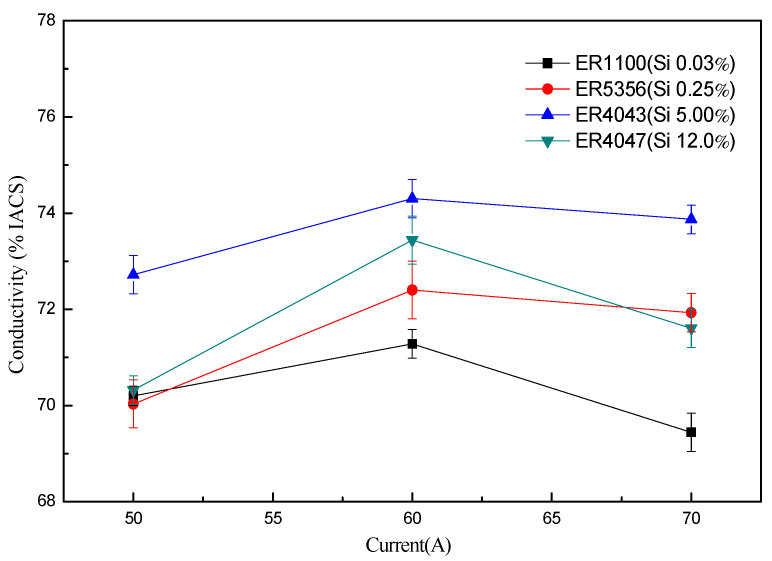
Conductivity of joints in different heat input.

As shown in Figure 9 and Figure 10, it can be found that the resistivity of joints with the same filler wires raised with the increase in heat input firstly, then, the value of resistivity decreased when the weld current continued to increase to a certain value, but just for the conductivity of those specimens, the opposite appeared. 

## 4. Discussion

### 4.1. Effect Mechanism of Alloy Elements on IMC Layer

From the analysis of the above results, it can be seen that the composition of intermetallic compounds was the same under the similar welding conditions, but the thickness was obviously different using different filler wires. The main reason for this phenomenon was that the alloy elements from the wire have an effect on the growth of intermetallic compounds. Furthermore, Si elements in the filler wires were able to discourage growth of intermetallic compounds, but Mg in the filler wires had taken no obvious effect. Hence, with the increase in the Si content, growth of intermetallic compounds became slower obviously. Through the analysis of alloy elements from filler wires, the best content of Si was 12%, which led to the action of discouraging growth more effectively.

In the welding seam, the mass fraction of Al elements was higher than that of Si elements; the Cu atom in the copper base was easy to diffuse in the welding seam when the bead was molten in liquid state, thus, it led to the range of contact interface between the Cu atom and Al atom becoming larger. Meanwhile, the IMC was generated in the interface between the Cu atom and Al atom. However, the atom radius of Si was smaller than that of Al; it needed less activation energy to diffuse. When the atom of Si obtained a small driving force, the Si atom spread and enriched together in the front of interface of intermetallic compounds, and it hindered the Al atom and Cu atom to transfer in the interface of intermetallic compounds.

The diffusion velocity of elements depended on the size of activity energy between the welding joints. Thus, it has been found that Si had an inhibitory effect on the growth. Owing to the content of Al in intermetallic compounds being higher than that of Si, Si atom diffusion in the aluminum substrate was mutual diffusion between the dissimilar metals and the Al atom was self-diffusion. On the one hand, the atom radius of Si was smaller than that of Al, resulting for less activation energy of Si atom diffusion; on the other hand, Si atom diffusion provided enough driving force, and the Si atom melted and migrated to the copper side under the effect of the arc heat and heat conduction on welding pool. At the same time, intermetallic compounds firstly were formed by Cu and Al atom in interface between aluminum and copper before the Si atom had not worked on that yet. Consequently, copper and aluminum atoms were restricted to migrate obviously, and the control of the growth of intermetallic compounds was realized [20,21,22].

**Figure 11:**
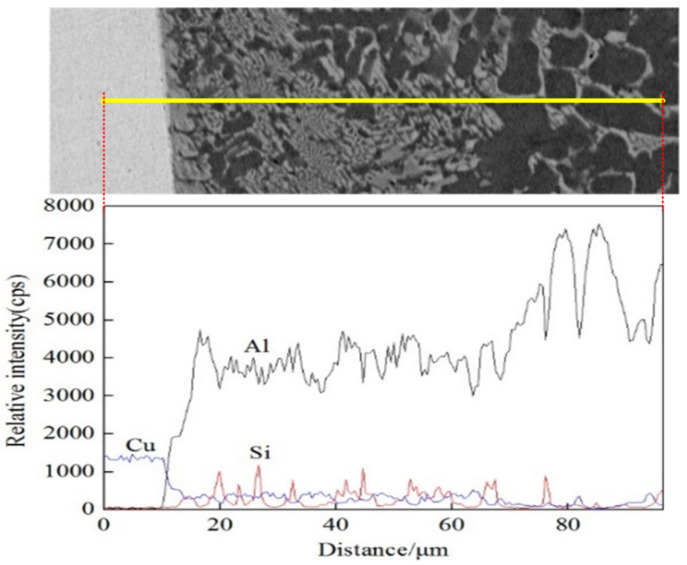
Linear scanning image of the brazing zone of joints at copper side with 4047 filler wires.

In order to further analyze the elements diffusion in the brazing area of aluminum and copper, especially the copper side, the joint filled by ER4047 filler wires was scanned with EDS. Figure 11 shows a linear scanning image of the brazing zone of joints at the copper side with 4047 filler wires; Al elements were up, but Cu elements were down, then, there was a platform suddenly. It was observed that the contents of aluminum and copper were distributed equally in the area where they were close to the copper side; the intermetallic compound began to form in this zone. Meanwhile, the Si elements almost were not pervaded to the Cu side, and they were concentrated on the area of the intermetallic compounds mostly.

### 4.2. Effect Mechanism of Alloy Elements on Tensile Strength

The differences of tensile strength was mainly related to its main alloy elements when adapting the similar welding parameters. Due to the addition of Si elements from filler wires, the flow of liquid in the molten pool became better, which can inhibit the formation of hot crack, and the Si elements can effectively inhibit the growth of brittle-hard Al_2_Cu phase. However, when the filler wires were ER4043 and ER4047, the change in strength was not obvious, there was a large amount of Si precipitating in copper side brazing interface zone, which may weaken the mechanical properties of joints.

### 4.3. Effect Mechanism of Alloy Elements on Microhardness

From the analysis of the previous results, it is found that the microhardness of joints was the highest when the content of Si elements was 12% in filler wires, there were more Si elements in filler wires, and the Si elements introduced the joints to form more Al–Si eutectic, which was closely related to the microhardness. Some scholars found that the more Al–Si eutectic was in the butt joints, the higher the microhardness of the butt joints was [23]. Therefore, adopting ER4047 filler wires, the microhardness increased obviously due to the influence of alloy elements Si. On the contrary, the microhardness of the joint was changed little by using Al–Mg welding wires.

### 4.4. Effect Mechanism of Alloy Elements on Conductivity

It can be concluded that there were many factors to influence the conductivity of joints [24,25,26], such as wettability, weld defects, intermetallic layer thickness, etc. Contrasting Table 8 and Table 11, it can be found that the resistivity of intermetallic compounds was higher than that of aluminum or copper base metal. The Al_2_Cu phase was easily generated in the seam and had a close relationship with the resistivity of the joint. Although its resistivity was the smallest among several Al–Cu intermetallic compounds, it was two times the resistivity of Al base and four times the Cu base. Thus, it can be seen that the intermetallic compound had a great effect on the electrical conductivity of the aluminum/copper brazing joints. Furthermore, the increase in intermetallic layer thickness caused the resistivity of joints raised and joints conductivity was opposite.

However, in a previous study [12], the results show that the thickness of intermetallic compounds and α(Al) + Al_2_Cu eutectic was improved with the increase in heat input, so it brings the conductivity of joints decreasing continually. However, it was observed from Figure 10 that the conductivity of joints was not a simple linear relationship from 50 to 70 A; it tended to increase firstly, then decreased, and increased again. It was explained that the appearance of joints was worse when the current of the main road was 50 A, the welding defect appeared, and the wettability of welding wire on the base metal surface were poor, this factor becoming the leading influence on the conductivity of joints. When the current of main road were 60 and 70 A, the sound appearance of the joints can be obtained, the leading factor was the thickness of intermetallic compounds and α(Al) + Al_2_Cu eutectic, and the higher the thickness of intermetallic compounds was, the lower the conductivity of joints was.

**Figure 12:**
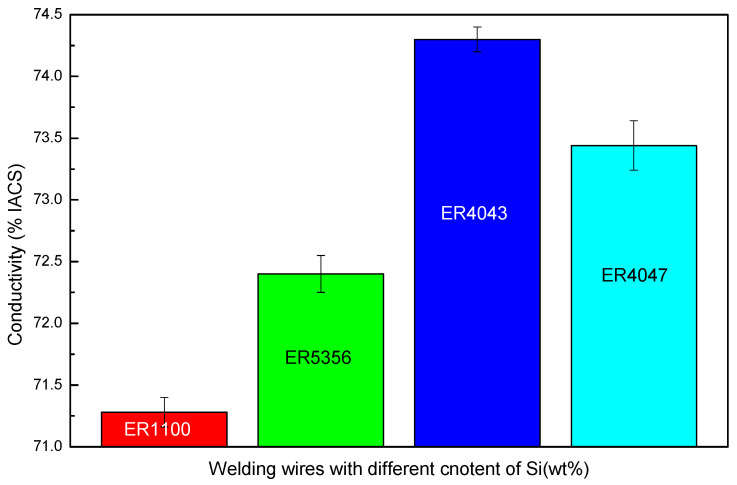
The change of conductivity of the butt joints with the increase in Si elements ($I_{main}=60\;A$).

As shown in Figure 12 ($I_{main}=60\;A$), the conductivity of joints using ER1100 was the least, meanwhile, the content of Si also was the least. It was found that with the increase in the content of Si obviously, the conductivity of joints was improved for using ER5356 and ER4043 filler wires. Although the Si content of ER4047 filler wires was more than two times that of ER4043 filler wires, the conductivity of joints using ER4047 was lower than that using ER4043. It was shown that the conductivity of joints increased first and then, decreased with the increase in Si content in the wires. The reason that caused this phenomenon was that the Si elements added first inhibit the formation of Al–Cu intermetallic compounds, which improved the conductivity of the joints. However, when the content of Si in the filler wires was too high, the Si phase was precipitated in the butt joints after the solidification of the liquid metal, and the Si elements were a semiconductor material and the resistivity was large, about 23 × 10^10^ Ω·m, which would seriously undermine the conductive performance of the joint. Therefore, the resistance of joints would be higher with ER4043 filler wires than that of the butt joints with the ER4047. 

## 5. Conclusions

(1) The pulsed DE-GMAW brazing technique was used with four types of filler wires and the microhardness of the weld seam was obviously improved when using the Al–Si filler wires. Moreover, comparing with the pure aluminum and Al–Mg filler wires, the tensile strength of the joints formed by Al–Si filler wires was higher; the Al–Si filler wires could be more suitable to improve the mechanical property of Al–Cu joints.

(2) The copper side brazing interface zone mainly consists of Al_2_Cu IMCs and eutectic Al–Cu. Due to diffusion velocity of elements in the front of the interface of intermetallic compounds, the thickness of the Al_2_Cu IMCs layer decreases with the increase in the Si content from Al–Sifiller wires into the Cu base metal; the minimum value was 3 µm. However, for the joints obtained with pure aluminum filler wire, the thickness of the intermetallic compound layer was about 20 µm.

(3) For the sound joints, the conductivity of joints with Al–Si filler wires was superior to others, and it was the worst when using pure Al filler wires. For the joints formed with Al–Sifiller wires, the high content of Si elements from filler wires could damage the conductivity of joints.

## Figures and Tables

**Table 1:** Chemical composition of filler wires (mass fraction, %).

Filler Wires	Si	Mg	Fe	Cu	Zn	Mn	Ti	Cr	Al
ER1100	0.03	-	0.18	0.002	0.013	0.003	-	-	Bal.
ER4043	5.00	0.05	0.80	0.30	0.10	0.05	0.20	-	Bal.
ER4047	12.00	0.10	0.80	0.30	0.20	0.15	-	-	Bal.
ER5356	0.25	5	0.5	0.05	0.05	0.15	0.1	0.15	Bal.

**Table 2:** Chemical composition of T2 copper (mass fraction, %).

Cu + Ag	Fe	Pb	Ni	Sb	S	As	Bi	Other Elements
≥99.9	≤0.005	≤0.005	≤0.005	≤0.002	≤0.005	≤0.002	≤0.001	0.06

**Table 3:** Chemical composition of 1060 pure Al (mass fraction, %).

Si	Cu	Mg	Zn	Mn	Cr	Fe	Al
0.25	0.10	2.2~2.8	0.10	0.10	0.15~0.35	0.40	Bal.

**Table 4:** Welding parameters.

The Types of Filler Wires	Average Current in Main Road (A)	Average Current in Bypass Road (A)	Argon Gas Flow in Main Road (L/min)	Argon Gas Flow in Bypass Road (L/min)	Pulse Frequency in Main and Bypass Road (Hz)
ER1100	50/60/70	25	20	5	80
ER5356	50/60/70	25	20	5	80
ER4043	50/60/70	25	20	5	80
ER4047	50/60/70	25	20	5	80

**Table 5:** Technical parameters of an intelligent metal conductor resistivity instrument.

The Project Name	Resistance	Resistivity	Conductivity
Measuring range	0.1 µΩ~150 Ω	0.01~2.5 µΩ·m	0.69%~172% International Annealed Copper Standard (IACS)
resolution ratio accuracy	0.1 µΩ (I = 1 A) ± 0.15%	10^−4^~10^−6^ µΩ·m	0.01~0.001% IACS

**Table 6:** EDS results in selected zone from Figure 5/at%.

Filler Wires	Points	Al	Cu	Si	Mg	Possible PHASE
ER1100	A	69.3	30.7	-	-	Al_2_Cu
	B	82.6	17.4	-	-	α(Al) + Al_2_Cu
ER5356	C	68.2	31.0	-	0.8	Al_2_Cu
	D	84.2	13.0	-	2.7	α(Al) + Al_2_Cu
ER4043	E	67.7	32	0.3	-	Al_2_Cu
	F	84.7	13.8	1.5	-	α(Al) + Al_2_Cu
ER4047	G	66.2	33.1	0.7	-	Al_2_Cu
	H	83.8	14.6	1.6	-	α(Al) + Al_2_Cu
	I	19.8	6.3	73.9	-	Si

**Table 7:** Tensile strength of samples.

Filler Wires	ER5356	ER4043	ER4047
Tensile strength	112.0	157.9	161.4

**Table 8:** Conductivity of base metal.

Material	Resistivity (Ω·m)	Conductivity (%IACS)
1060 pure aluminum plate	2.84 × 10^−8^	60.69
T2 copper	1.65 × 10^−8^	104.7

**Table 9:** Physical and mechanical properties of pure aluminum and T2 copper [18,19].

Material	Melting Point (°C)	Thermal Conductivity W/(m °C)	Resistivity (Ω·m)	Tensile Strength (MPa)
1060 pure aluminum	660	218	2.9 × 10^−8^	60
plate T2 copper	1083	391	1.7 × 10^−8^	195

**Table 10:** Electrical conductivity of aluminum/copper melt welding seam.

Filler Wires	Average Current in Main Road (A)	Resistance/µΩ	Resistivity/µΩ·cm	Conductivity/%IACS
ER1100	50	122.80	2.46	70.20
ER1100	60	120.94	2.42	71.28
ER1100	70	124.14	2.48	69.44
ER5356	50	123.10	2.46	70.03
ER5356	60	119.07	2.38	72.40
ER5356	70	119.85	2.40	71.93
ER4043	50	118.54	2.37	72.72
ER4043	60	116.02	2.32	74.30
ER4043	70	116.70	2.33	73.87
ER4047	50	122.59	2.45	70.32
ER4047	60	117.38	2.35	73.44
ER4047	70	120.40	2.41	71.60

**Table 11:** Resistivity of aluminum/copper intermetallic compounds (data from reference [27]).

Symbol	Composition	Al (wt %)	Cu (wt %)	Resistivity (Ω·m)
δ	Cu_3_Al_2_	22	78	13.4 × 10^−8^
ζ_2_	Cu_4_Al_3_	25	75	12.2 × 10^−8^
η_2_	CuAl	30	70	11.4 × 10^−8^
θ	CuAl_2_	45	55	8.0 × 10^−8^
